# RedMan-GreenMan: Co-Designed Pedestrian Safety Game Prototype for Children With Autism

**DOI:** 10.2196/69260

**Published:** 2026-01-15

**Authors:** Jason Carter Stanton, Nicole L Peel, Caroline J Mills, Paul P Breen

**Affiliations:** 1 The MARCS Institute for Brain, Behaviour & Development, Western Sydney University, 160 Hawkesbury Road, Westmead, NSW 2145, Australia, 61 452197751; 2 School of Health Sciences, Western Sydney University, Campbelltown, Australia; 3 Translational Health Research Institute, Western Sydney University, Westmead, Australia

**Keywords:** autism, game, pedestrian, safety, training

## Abstract

This letter presents the conceptualization, design, and technical evaluation of the RedMan-GreenMan game co-designed with carers, aimed to help children with autism spectrum disorder (hereafter autism) acquire pedestrian safety skills. While the system has been implemented and is in active use, no empirical evaluation of learning outcomes or behavioral impact has been conducted to date, and the focus of this work is on system development, functionality, and technical evaluation.

## Introduction

Children with autism face a higher risk of unintentional injury and death compared to their peers, as challenges with attention, motor control, and cognitive delays in some children can hinder the development of essential safety skills [1,2]. In collaboration with the Skillz4me Family Centre for Disabilities, a not-for-profit organization established to support children with moderate to profound disabilities, it was identified that while some research has been completed in the area [3-5], there are currently no pedestrian road safety programs available that adequately address the specific needs of children with autism.

A specific barrier for children with autism is the difficulty in forming clear associations between related objects [6,7]. For example, a small toy train may not be readily associated with an actual full-sized train. To address this challenge, we co-designed a simple game, RedMan-GreenMan, which importantly integrates a real pedestrian traffic signal. This approach may increase the likelihood of recognition and association by the child, and in turn may support the potential transfer of safety skills from the indoor space and gameplay to the public, real-world environment.

An additional design consideration was the integration of a performance recording system to enable staff to monitor each child’s interaction with the game over time.

This paper describes the design and in situ testing of the RedMan-GreenMan system. Testing aimed to validate the technical aspects of the system; it did not include any assessment of children, pedagogical outcomes, or effectiveness of the intervention in teaching safety skills.

## Methods

### User Requirements

Scope for the design, development, and commissioning of the system was established. Briefly, the user requirements were:

- Use of a real pedestrian traffic light;
- Remote controllable operation of the game;
- Random timing of gameplay in operation, to prevent inadvertent signaling from staff; and
- Date-time stamping of in-game child response.

### System Requirements

**Figure 1.**
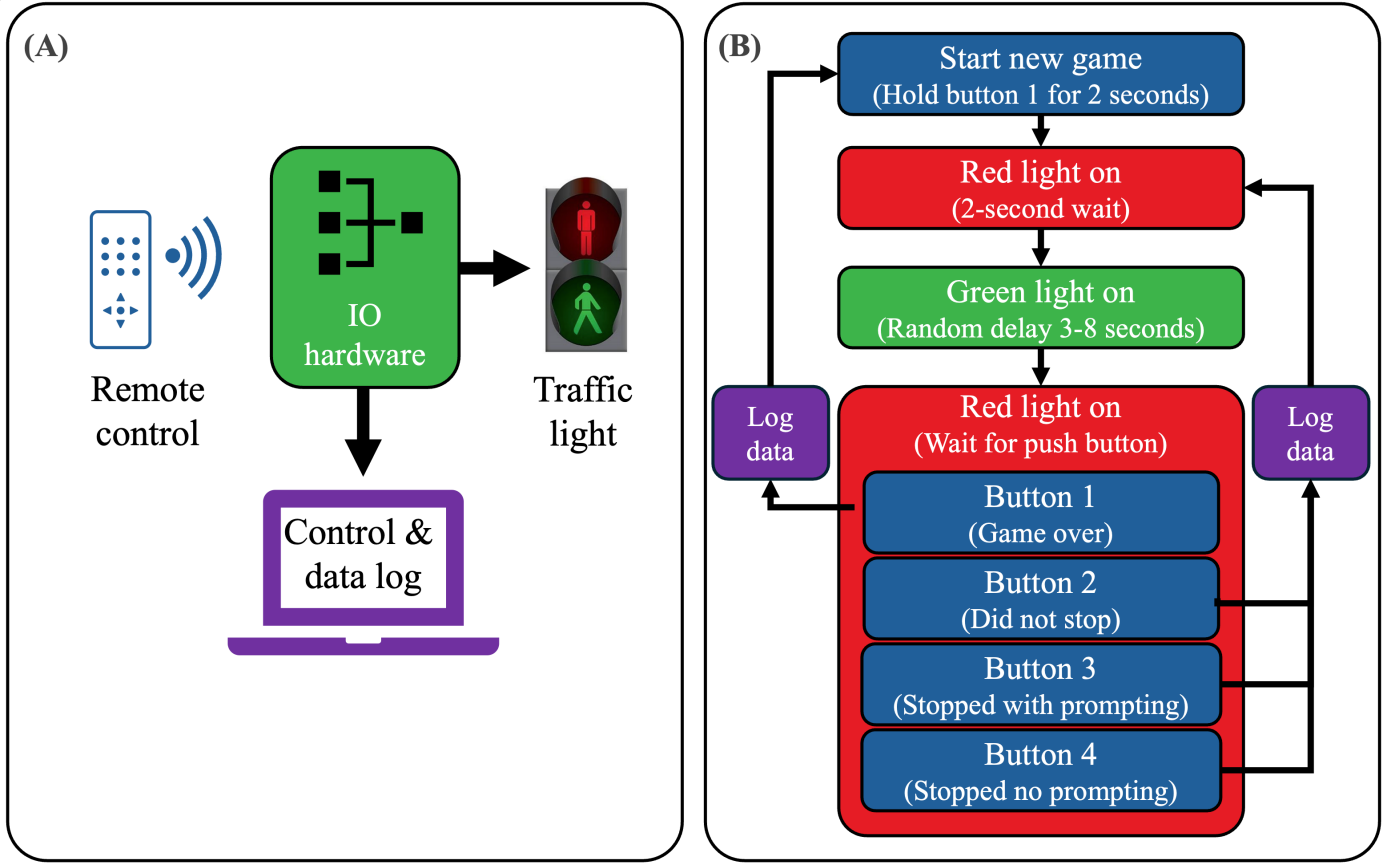
(A) System topology and (B) process control developed for RedMan-GreenMan. Key requirements included a need for the system to be controllable by a remote control to allow the staff member to interact with children within the center. The categorization of child responses was intentionally limited to three options to simplify decision-making for the busy staff member.

User requirements led to the system topology and process control as shown in Figure 1.

### System Evaluation

A series of experiments was conducted to rigorously evaluate system functionality, accuracy, precision, and operational limits.

- System functionality during repeated operation cycles: With the remote positioned 5 meters from the receiver, the program was initiated by pressing and holding the “1” key for 2 seconds. A fixed button selection pattern (2-3-4-3-2) was executed and terminated by pressing the “1” key. This cycle was repeated 40 times to assess system reliability and accuracy under consistent operational conditions.
- Traffic light timing verification: Traffic light change periods were logged to a file and cross-referenced with video recordings for validation. The accuracy and precision of randomly timed light changes (range 3‐8 s) were analyzed.
- System reliability at increasing range: System reliability was tested with 30 traffic light changes at a 2-meter distance between the remote and receiver. This process was repeated at 5-meter intervals, gradually increasing the distance until complete communication failure was observed.

A single device was used in all testing within the Skillz4me Family Centre for Disabilities with direct line of sight between the remote control and receiver up to a range of 75 m. Distances greater than 75 m lost the line of sight, as measurements were taken in the foyer (80‐85 m), entry area (90 m), and outside the building (≥95 m).

### Ethical Considerations

Ethical review was not sought as no human subjects were involved in the research.

## Results

**Figure 2.**
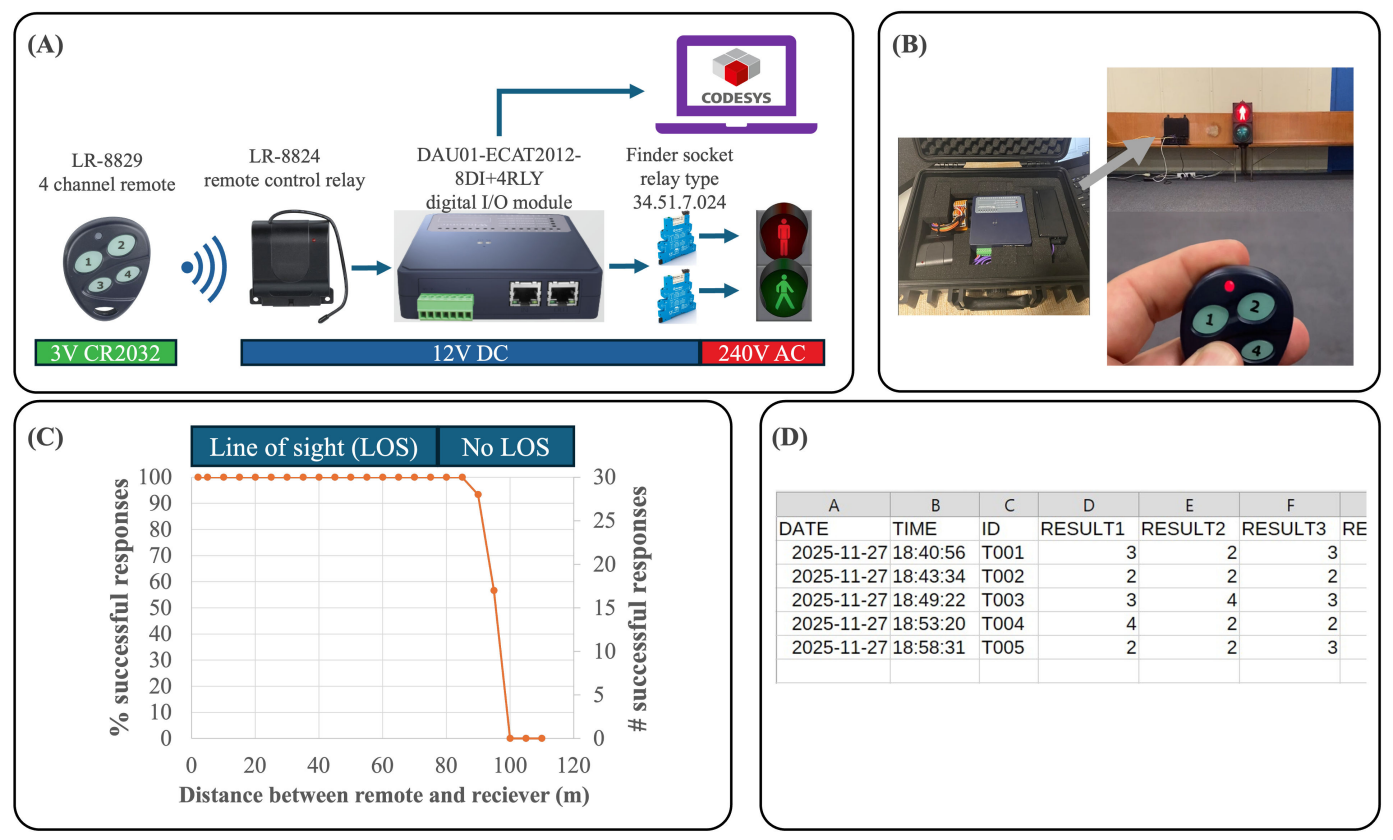
(A) Developed system architecture showing specific components and power requirements. (B) Actual system created: gray arrow points to system electronics next to traffic light. (C) Reliability of system response at increasing range; in order to reach failure, the researcher had to exit the center. (D) Demonstration of a system datalog for 5 consecutive simulated games where the monitored individual (ID) is different for each game (no human subjects involved).

An image of the developed system and its architecture is shown in Figure 2. The system’s performance was evaluated through three distinct experiments. In experiment 1, a predefined sequence pattern was repeated 40 times, achieving 100% accuracy and demonstrating exceptional reliability during repeated operational cycles.

Experiment 2 focused on the accuracy of the traffic light timing, defined as the mean difference between measurements and logged reference values, which was found to be 0.096 seconds, indicating a very slight positive bias. The precision, assessed as the SD of residuals (differences between the secondary measurements and their corresponding references), was 0.072 seconds, reflecting consistent measurement performance.

Experiment 3 assessed the system’s reliability over increasing distances. The system operated with 100% reliability up to a range of 85 m; this includes 10 m (from 75 to 85 m) without a direct line of sight. Beyond this range, performance declined sharply, culminating in total failure at 100 meters. It is important to note that the maximum effective range was constrained by indoor physical limitations, as direct line of sight was obstructed by the layout of the testing environment (Figure 2C). These results highlight the system’s robustness and accuracy under controlled conditions while identifying potential limitations in extended-range applications.

## Discussion

This study presents the conceptualization, design, and technical evaluation of RedMan-GreenMan, a gamified intervention co-developed with the Skillz4me Family Centre for Disabilities. The system was designed to support children with autism in acquiring pedestrian safety skills by addressing challenges in associative learning. By incorporating an authentic pedestrian traffic signal, the prototype aims to bridge the perceptual and cognitive divide between the virtual learning environment and real-world traffic scenarios.

Importantly, while the system has been implemented in accordance with user-defined requirements and is currently deployed operationally at the Skillz4me Family Centre for Disabilities, no human data were collected or analyzed for this study, and no educational or pedagogical evaluation has been conducted. The current work should not be interpreted as evidence of learning impact or behavioral change in the target population.

The system’s technical functionality has been validated in situ, but no empirical data is available regarding its effectiveness in teaching safety skills or supporting skill transfer to real-world contexts. Other limitations include the lack of scalability and portability of the prototype, and its construction remains cost-prohibitive (~US $350 for parts excluding laptop).

Future research should include structured educational trials to rigorously evaluate learning outcomes, skill retention, and transfer to real-world naturalistic settings. Such studies may benefit from mixed methods approaches, including behavioral observations, performance tracking, and input from caregivers or therapists. Parallel system development efforts should focus on improving affordability and portability, with the goal of enabling broader use in diverse educational and therapeutic settings.

## Supplementary material

10.2196/69260 Multimedia Appendix 1 System hardware design, bill of materials, and software.
