# Supplementary material for: RedMan-GreenMan: Co-Designed Pedestrian Safety Game Prototype for Children With Autism
Source: JMIR Serious Games. 2026 Jan 15;14:e69260. doi: 10.2196/69260 (PMC12806593; doi:10.2196/69260)
Supplement: Multimedia Appendix 1 [file games-v14-e69260-s001.pdf]

Appendix

Figure 2a in the manuscipt shows the developed system topology. The operator input to the control system is via a remote control. The outputs of the control system are the pedestrian traffic signals and data saved in CSV file format. Table 1 provides the bill of materials used to construct the system. Electrical schematics for power and input/output wiring are provided in Figures 1-3. The system software process flowchart is given in Figure 4, followed by the CODESYS program code and function blocks required to replicate the system. Program files are available at the following github repository https://github.com/superbreen/RedMan-GreenMan.git.

Table 1. Bill of Materials

Item	Description
Remote Control Transmitter/Receiver	LR-8829 & LR-8824
ETHERCAT IO Module	WINSONIC DAU01-ECAT2012-8DI+4RLY
Interface Relays	Finder Socket 93.01.0.024<br>Relay Type 34.51.7.024
Power Supply	MP3242 12V 5A DC
Traffic Light	AC Traffic Signal – Local Model

Electrical Schematics

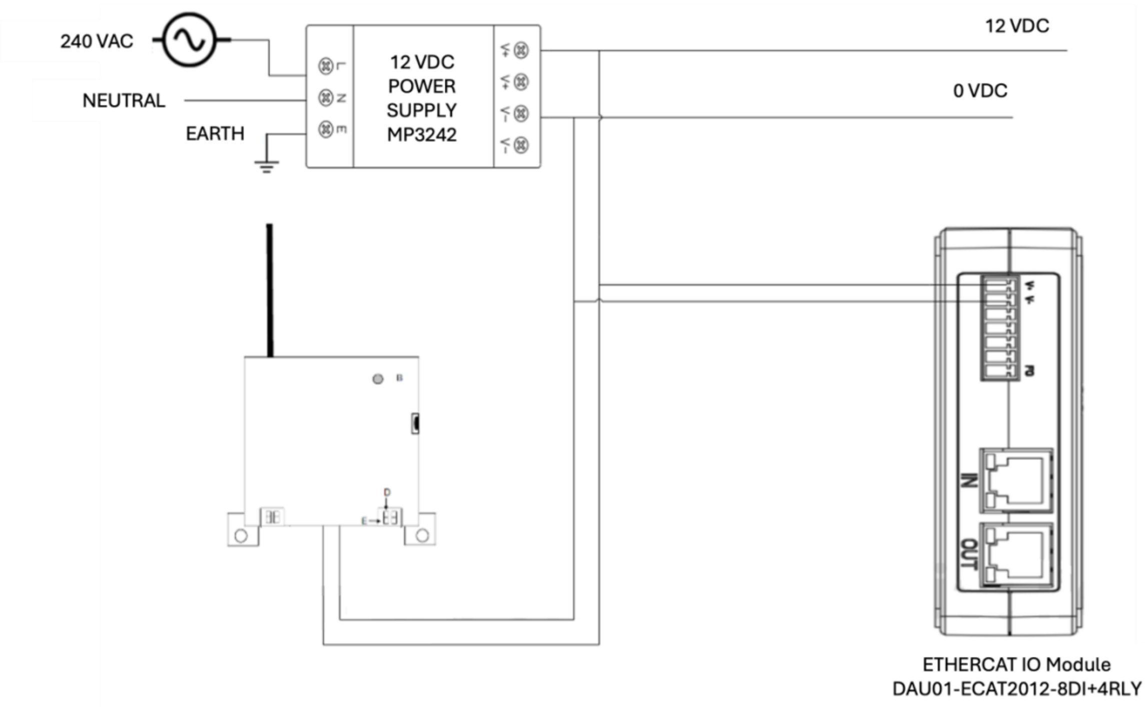

Figure 1. System Input Power Supply

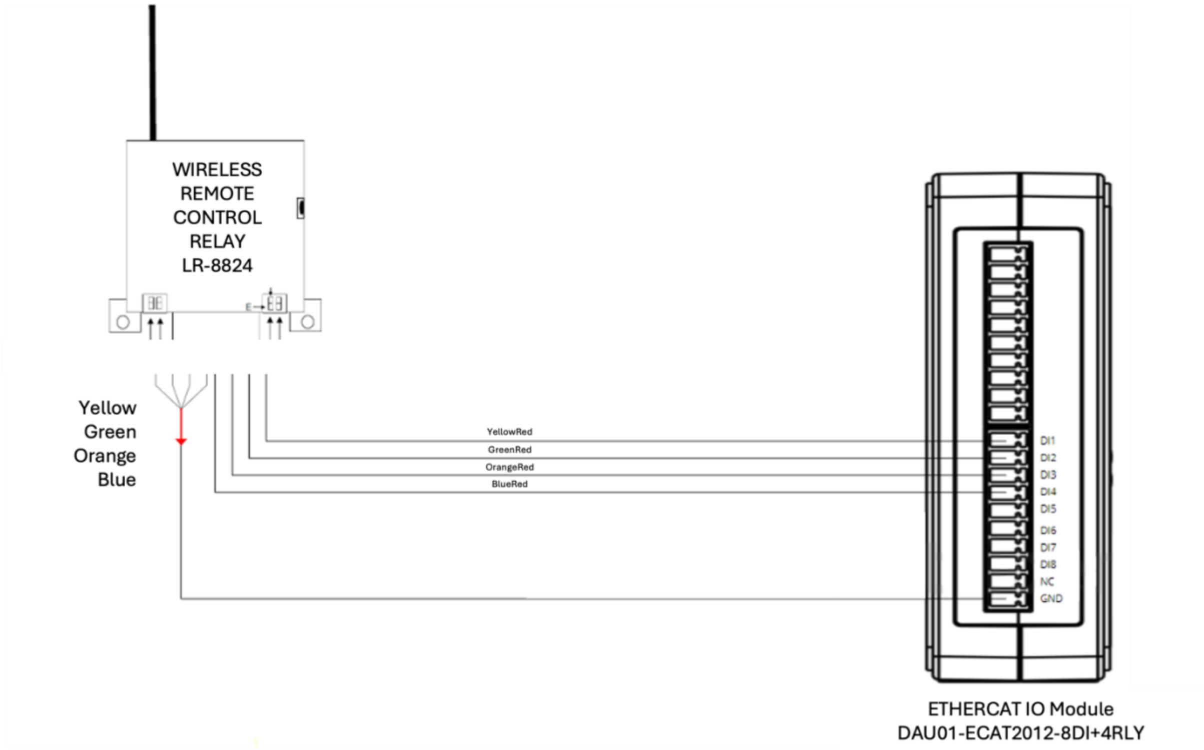

Figure 2. System Input Wiring

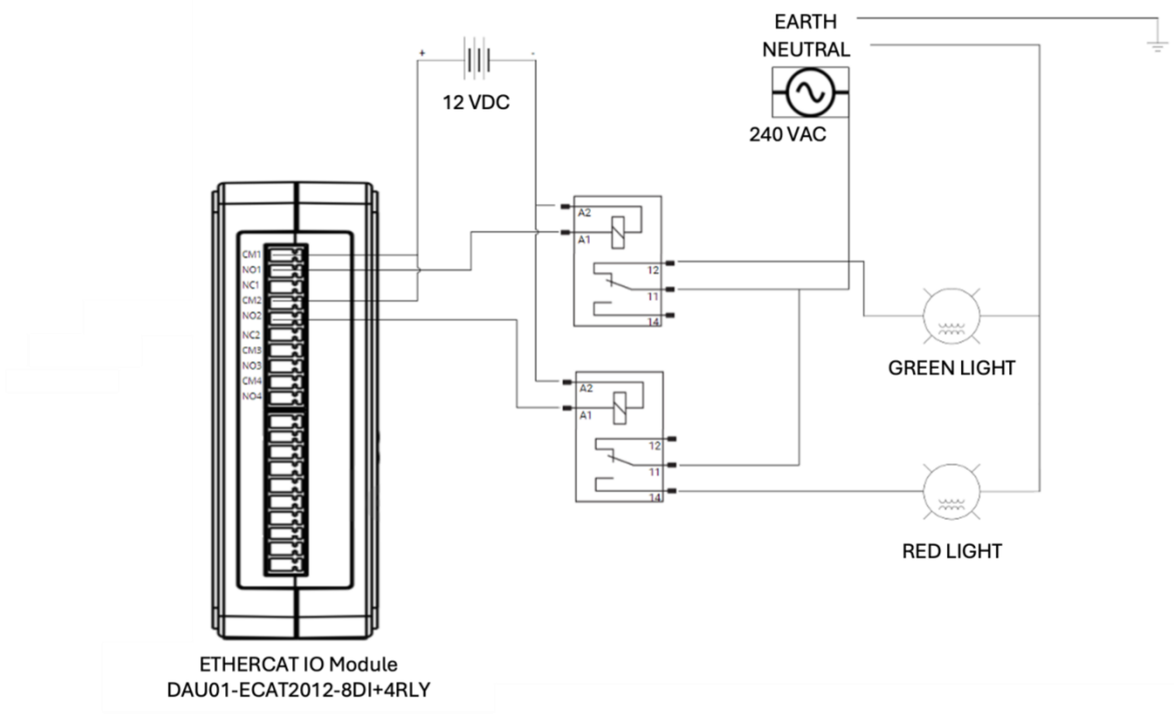

Figure 3. System Output Wiring and Power Supply

System Software Process Flowchart

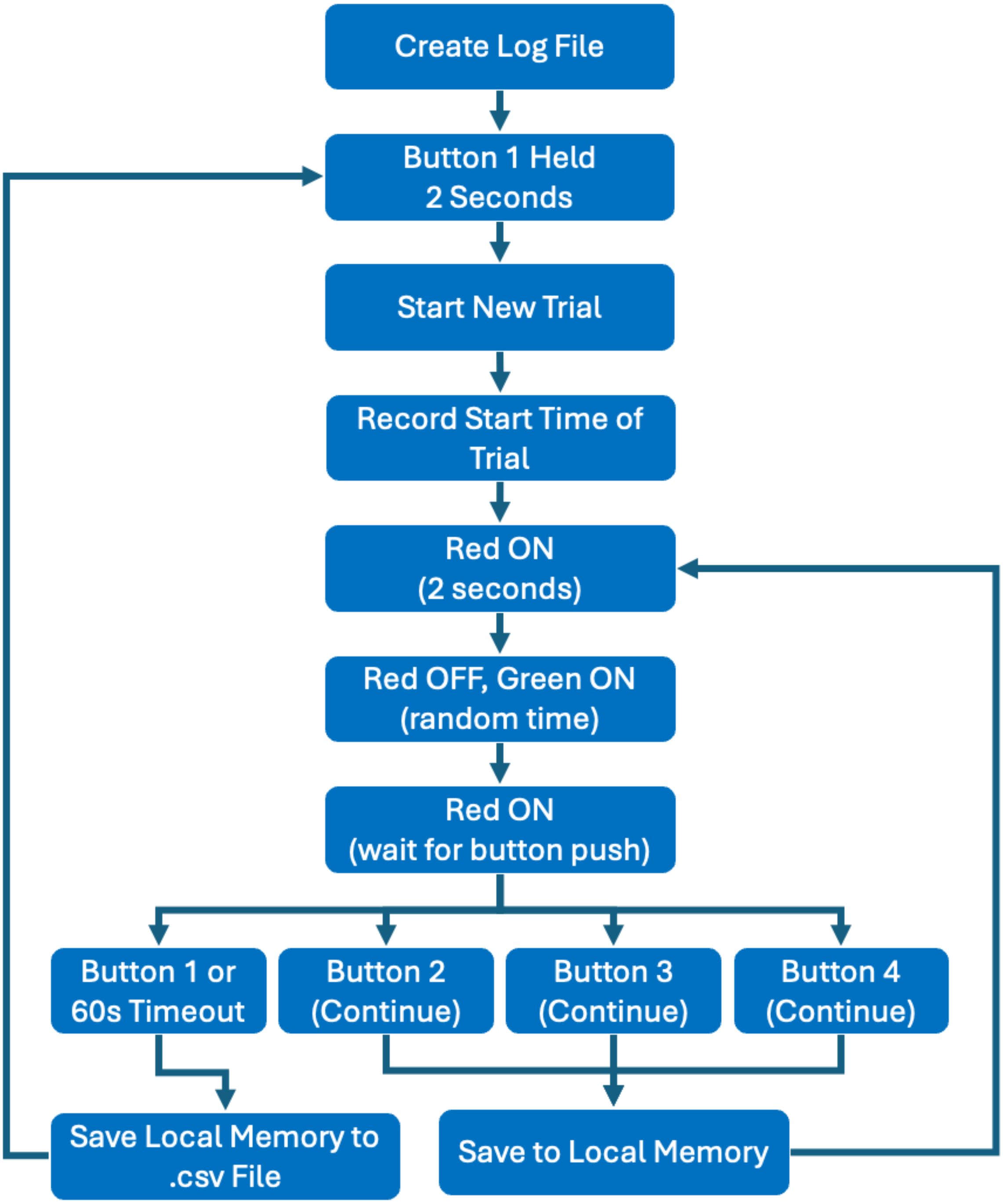

Figure 4. High Level Software Process Flowchart.

System Software (CODESYS)

```
PROGRAM prg_Main

VAR
	// Variable Definitions
	iStep				: INT;
	// Program is step (or state) based using this variable
	iCycle				: INT;
	// Cycle number. Added to the results file
	sCurrentMessage			: STRING(255);
	// Local variable used for building up the record that will be written to file
	sFinalMessageToWrite		: STRING(255);
	// The final record that is written to the logfile
	sLogFilePathAndName		: STRING(255):= 'C:\Experiment1_LogFile.csv';
	// Headings used in logfile
	sLogFileFirstLine		: STRING(255) :=
	'DATE,TIME,ID,RESULT1,RESULT2,RESULT3,RESULT4,RESULT5,RESULT6,$r$n';
	tRandomTime			: TIME := T#2S;
	// Variable for random time interval for GreenLight ON
	sRandomTime			: STRING;
	// The random time as a string for writing to Logfile
END_VAR

VAR
	// Function Block Calls
	TON_Timer			: TON; // IEC61331 Standard Timer Function Block
	fbWriteToFile			: fb_FileWrite; // Function Block for creating a file
	fbAppendToFile			: fb_FileAppend;
	// Function Block for writing to an existing file
	fbRandom3to8			: fb_RandomInt;
	// Function Block to generate a random integer
END_VAR

CASE iStep OF
00: // Create a new file Once Only
	iCycle := 0;
	fbWriteToFile(xStart:=TRUE, sContentWrite:=sLogFileFirstLine,
	sFileNameAndPath:=sLogFilePathAndName);
	IF	(fbWriteToFile.xDone) THEN
		fbWriteToFile(xStart:=FALSE);
		iStep := 05;
	END_IF

05:	// Wait for Button1 pressed & held for 2 seconds to start a new sequence
	xLightGreen_DQ1 := TRUE;
	xLightRed_DQ2 := TRUE;
	IF (NOT TON_Timer.Q) THEN
		TON_Timer(IN:=xButton1_DI1, PT:=T#2S);
```

```
ELSE
        TON_Timer(IN:=FALSE);
        xLightGreen_DQ1 := FALSE;
        xLightRed_DQ2 := FALSE;
        iStep := 10;
END_IF
// Reset variables
sFinalMessageToWrite := '';
sCurrentMessage := '';
fbAppendToFile(xStart:=FALSE);

10:     // Wait for all buttons to be released before continuing
        IF (NOT xButton1_DI1) AND (NOT xButton2_DI2) AND (NOT xButton3_DI3) AND
        (NOT xButton4_DI4) THEN
                iStep := 15;
        END_IF

15:     // Add the Date, Time and Iteration of the Trial to the message. This is written to
        file at end of the test cycle.
        iCycle := iCycle + 1; // increment cycle number
        sCurrentMessage := fun_CONCAT5(g_sCurrentDate, ',', g_sCurrentTOD, ',',
        fun_MakeTestNumber(iCycle));
        iStep := 20;

20:     // RED LIGHT is ON for 2 seconds before progressing with sequence, no buttons
        can be pressed to progress.
        xLightGreen_DQ1 := FALSE;
        xLightRed_DQ2 := TRUE;
        IF (NOT TON_Timer.Q) THEN
                TON_Timer(IN:=TRUE, PT:=T#2S);
        ELSE
                IF (NOT xButton1_DI1) AND (NOT xButton2_DI2) AND (NOT
                xButton3_DI3) AND (NOT xButton4_DI4) THEN
                        TON_Timer(IN:=FALSE);
                        fbRandom3to8(xGenerateRDM:=FALSE);
                        // Ensure the Random Function Block is Reset
                        iStep := 25;
                END_IF
        END_IF

25:     // Generate a Random Time for the Green Light duration
        fbRandom3to8(xGenerateRDM:=TRUE, xMin:=3, xMax:=8);
        tRandomTime := INT_TO_TIME(fbRandom3to8.rdmOut*1000);
        sRandomTime := INT_TO_STRING(fbRandom3to8.rdmOut);
        iStep := 30;

30:     // GREEN LIGHT is ON, wait random time
        xLightGreen_DQ1 := TRUE;
        xLightRed_DQ2 := FALSE;
```

```
        IF (NOT TON_Timer.Q) THEN
            TON_Timer(IN:=TRUE, PT:=tRandomTime);
        ELSE
            TON_Timer(IN:=FALSE);
            iStep := 35;
        END_IF

35:     // RED LIGHT is ON, wait for button press or TIMEOUT
        xLightGreen_DQ1 := FALSE;
        xLightRed_DQ2 := TRUE;
        // Log Button Presses
        IF (xButton1_DI1) THEN
            // Button 1 Pressed. Sequence Finished
            sFinalMessageToWrite := fun_CONCAT4(sCurrentMessage, ',',
            g_sCurrentTOD, ',$r$n');
            iStep := 40;
        ELSIF (xButton2_DI2) THEN
            // Button 2 Pressed
            sCurrentMessage := CONCAT(sCurrentMessage, ',2');
            iStep := 20;
        ELSIF (xButton3_DI3) THEN
            // Button 3 Pressed
            sCurrentMessage := CONCAT(sCurrentMessage, ',3');
            iStep := 20;
        ELSIF (xButton4_DI4) THEN
            sCurrentMessage := CONCAT(sCurrentMessage, ',4');
            iStep := 20;
        ELSE
            // Timeout after 60 seconds
            IF (NOT TON_Timer.Q) THEN
                TON_Timer(IN:=TRUE, PT:=T#60S);
            ELSE
                TON_Timer(IN:=FALSE);
                sFinalMessageToWrite := CONCAT(sCurrentMessage,
                ',TimeOut,$r$n');
                iStep := 40;
            END_IF
        END_IF

40:     // Append to File
        fbAppendToFile(xStart:=TRUE, sContentWrite:=sFinalMessageToWrite,
        sFileNameAndPath:=sLogFilePathAndName);
        IF (fbAppendToFile.xDone) THEN
            fbAppendToFile(xStart:=FALSE);
            iStep := 05;
        END_IF;
END_CASE
```

```
PROGRAM prg_DateTime

VAR
	Result			: SysTypes.RTS_IEC_RESULT;
	eWeekDay		: UTIL.WEEKDAY;
END_VAR

// Get TimeStamp
dwTime1 := SysTimeRtc.SysTimeRtcGet(Result);
IF (Result = CmpErrors.Errors.ERR_OK) THEN
	SysTimeRtc.SysTimeRtcConvertUtcToLocal(dwTime1,dwTimeLocal);
END_IF

// Convert the DWORD into DATE-TIME and then DATE & TIME
g_dtCurrentDT := DWORD_TO_DT(dwTimeLocal);
g_datCurrentDate := DT_TO_DATE(g_dtCurrentDT);
g_todCurrentTime := DT_TO_TOD(g_dtCurrentDT);
g_tCurrentTime := TOD_TO_TIME(g_todCurrentTime);

// Convert date & time to STRING
g_sCurrentDate := MID(DT_TO_STRING(g_dtCurrentDT),10,4);
g_sCurrentTOD := MID(DT_TO_STRING(g_dtCurrentDT),8,15);
g_sCurrentHour := MID(DT_TO_STRING(g_dtCurrentDT),2,15);
g_sCurrentMinute := MID(DT_TO_STRING(g_dtCurrentDT),2,18);
g_sCurrentSecond := MID(DT_TO_STRING(g_dtCurrentDT),2,21);

// Convert date & time for FILE NAME
g_sFileNameDT := fun_CONCAT7(g_sCurrentDate, '_',g_sCurrentHour, '.',
g_sCurrentMinute, '.', g_sCurrentSecond);

// Convert the Weekday to a STRING for Visual display
eWeekDay := UTIL.DayOfWeek(g_datCurrentDate);
IF (eWeekDay = 1) THEN g_sWeekDay := 'Monday';
ELSIF (eWeekDay = 2) THEN g_sWeekDay := 'Tuesday';
ELSIF (eWeekDay = 3) THEN g_sWeekDay := 'Wednesday';
ELSIF (eWeekDay = 4) THEN g_sWeekDay := 'Thursday';
ELSIF (eWeekDay = 5) THEN g_sWeekDay := 'Friday';
ELSIF (eWeekDay = 6) THEN g_sWeekDay := 'Saturday';
ELSIF (eWeekDay = 7) THEN g_sWeekDay := 'Sunday';
ELSE g_sWeekDay := '--';
END_IF
```

```
FUNCTION_BLOCK fb_RandomInt

VAR_INPUT
        xGenerateRDM        :BOOL;
        xMin                :INT:=0;
        xMax:INT            :=100;
END_VAR

VAR_OUTPUT
        rdmOut              :INT;
END_VAR

VAR
        R_TRIG_0            : R_TRIG;
END_VAR
```

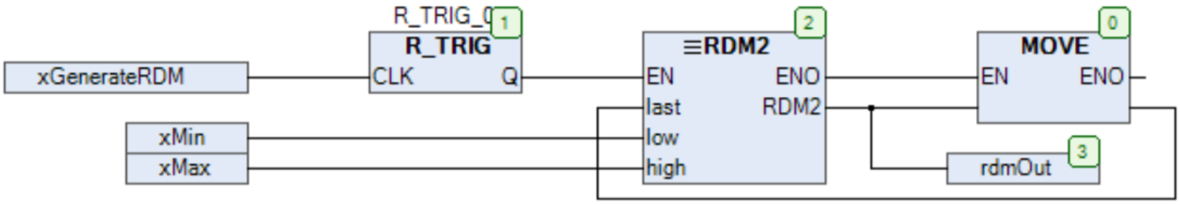

Figure 5: RedMan-GreenMan Random Function Block.

```
FUNCTION_BLOCK fb_FileWrite

VAR_INPUT
	xStart			: BOOL;
	// Set TRUE to write and read the demo file 'CFCTestfile.txt'
	sContentWrite		: STRING(255) := 'Codesys Hello World';
	// File content to write
	sFileNameAndPath	: STRING(255) := 'c:\Testfile.txt';
END_VAR

VAR
	Open_0			: FILE.Open;
	Write_0			: FILE.Write;
	Close_0			: FILE.Close;
END_VAR

VAR_OUTPUT
	xDone			: BOOL;
END_VAR
```

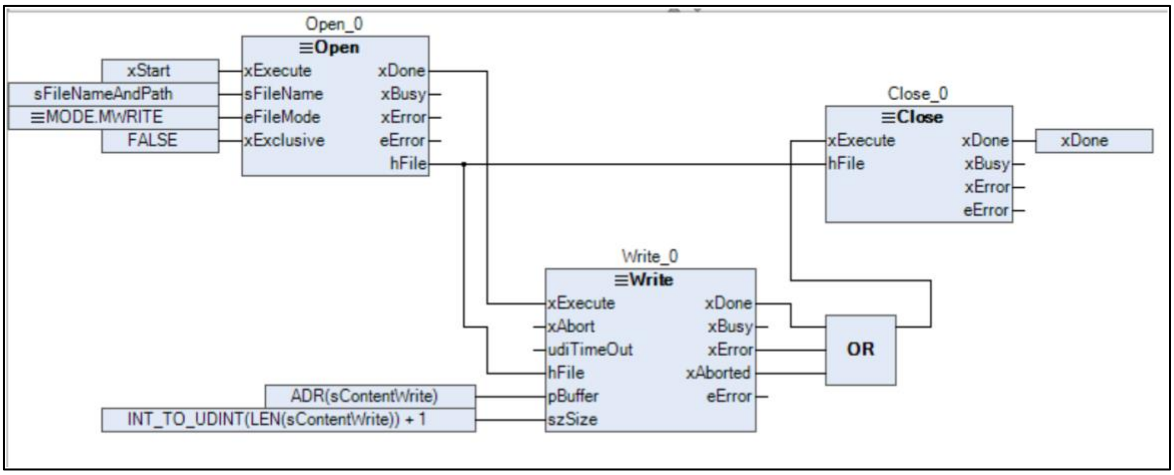

Figure 6: File Write Function Block

```
FUNCTION_BLOCK fb_FileAppend

VAR_INPUT
	xStart			: BOOL;
	// Set TRUE to write and read the demo file 'CFCTestfile.txt'
	sContentWrite		: STRING(255) := 'Codesys Hello World';
	// File content to write
	sFileNameAndPath	: STRING(255) := 'c:\Testfile.txt';
END_VAR

VAR
	Open_0			: FILE.Open;
	Write_0			: FILE.Write;
	Close_0			: FILE.Close;
END_VAR

VAR_OUTPUT
	xDone		: BOOL;
END_VAR
```

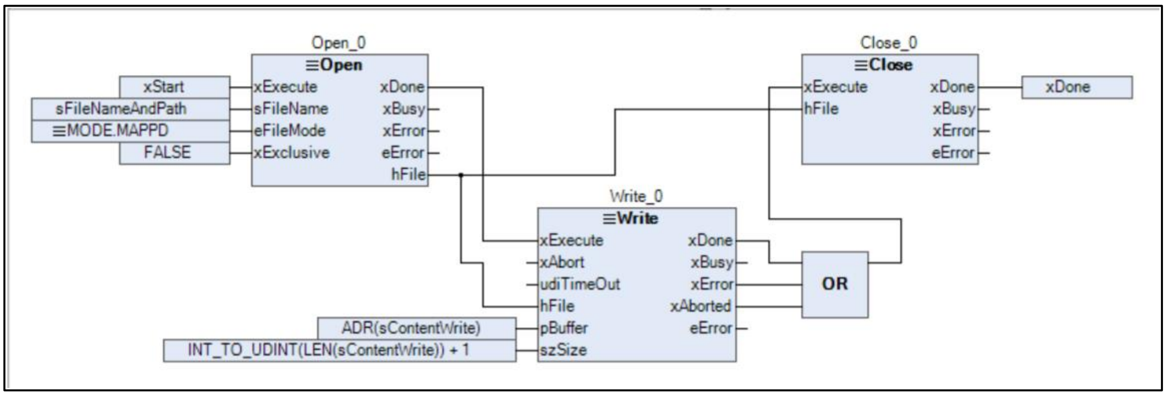

Figure 7: FileAppend Function Block.

```
VAR_GLOBAL
	// DateTime Variables
	dwTime1				: DWORD;
	dwTimeLocal			: DWORD;
	g_dtCurrentDT			: DT;
	g_datCurrentDate		: DATE;
	g_todCurrentTime		: TOD;
	g_tCurrentTime			: TIME;
	g_sCurrentDate			: STRING;
	g_sCurrentTOD			: STRING;
	g_sCurrentHour			: STRING;
	g_sCurrentMinute		: STRING;
	g_sCurrentSecond		: STRING;
	g_sWeekDay			: STRING;
	g_sFileNameDT			: STRING(255);
END_VAR

FUNCTION fun_CONCAT7 : STRING(255)
VAR_INPUT
	String1					: STRING(255);
	String2					: STRING(255);
	String3					: STRING(255);
	String4					: STRING(255);
	String5					: STRING(255);
	String6					: STRING(255);
	String7					: STRING(255);
END_VAR

fun_CONCAT7 :=
CONCAT(fun_CONCAT6(String1,String2,String3,String4,String5,String6),String7);
```

```
FUNCTION fun_MakeTestNumber : STRING

VAR_INPUT
	iNumber				: INT;
END_VAR

VAR
	sNumber				: STRING;
	iNumberOfCharacters		: INT;
	sResultString			: STRING;
END_VAR

sNumber := INT_TO_STRING(iNumber);
iNumberOfCharacters := LEN(sNumber);

CASE iNumberOfCharacters OF
	1: sResultString := CONCAT('T00',sNumber);
	2: sResultString := CONCAT('T0',sNumber);
	3: sResultString := CONCAT('T',sNumber);
ELSE
	sResultString := 'STR_error';
END_CASE

fun_MakeTestNumber := sResultString;
```
